# Modified international e-Delphi survey to define healthcare professional competencies for working with teenagers and young adults with cancer

**DOI:** 10.1136/bmjopen-2016-011361

**Published:** 2016-05-02

**Authors:** Rachel M Taylor, Richard G Feltbower, Natasha Aslam, Rosalind Raine, Jeremy S Whelan, Faith Gibson

**Affiliations:** 1 NIHR University College London Hospitals Biomedical Research Centre, UCL Hospitals NHS Foundation Trust, London, UK; 2 School of Health and Social Care, London South Bank University, London, UK; 3 Division of Epidemiology & Biostatistics, School of Medicine, University of Leeds, Leeds, UK; 4 Department of Applied Health Research, University College London, London, UK; 5 Centre for Outcomes and Experience Research in Children's Health, Illness and Disability, Great Ormond Street Hospital for Children NHS Foundation Trust, London, UK

**Keywords:** Teenagers and young adults, cancer, multi-disciplinary team, competence, BRIGHTLIGHT

## Abstract

**Objectives:**

To provide international consensus on the competencies required by healthcare professionals in order to provide specialist care for teenagers and young adults (TYA) with cancer.

**Design:**

Modified e-Delphi survey.

**Setting:**

International, multicentre study.

**Participants:**

Experts were defined as professionals having worked in TYA cancer care for more than 12 months. They were identified through publications and professional organisations.

**Methods:**

Round 1, developed from a previous qualitative study, included 87 closed-ended questions with responses on a nine-point Likert scale and further open-ended responses to identify other skills, knowledge and attitudes. Round 2 contained only items with no consensus in round 1 and suggestions of additional items of competency. Consensus was defined as a median score ranging from 7 to 9 and strength of agreement using mean absolute deviation of the median.

**Results:**

A total of 179 registered to be members of the expert panel; valid responses were available from 158 (88%) in round 1 and 136/158 (86%) in round 2. The majority of participants were nurses (35%) or doctors (39%) from Europe (55%) or North America (35%). All 87 items in round 1 reached consensus with an additional 15 items identified for round 2, which also reached consensus. The strength of agreement was mostly high for statements. The areas of competence rated most important were agreed to be: ‘Identify the impact of disease on young people's life’ (skill), ‘Know about side effects of treatment and how this might be different to those experienced by children or older adults’ (knowledge), ‘Honesty’ (attitude) and ‘Listen to young people's concerns’ (aspect of communication).

**Conclusions:**

Given the high degree of consensus, this list of competencies should influence education curriculum, professional development and inform workforce planning. Variation in strength of agreement for some competencies between professional groups should be explored further in pursuit of effective multidisciplinary team working.

**Strengths and limitations of this study**

- Our survey included representation from the range of professionals comprising the multidisciplinary team.
- We involved an international panel of experts.
- Our survey was presented in the English language which may have prevented some individuals we contacted from participating.
- We relied on international professional organisations to circulate the link to the survey; this may have biased our responses to particular professional group or nationality.

## Introduction

Teenage and young adult (TYA) medicine has emerged as a distinct speciality in healthcare, acknowledging and addressing the core tasks required to enable a young person to transition from childhood to adulthood.^1^ This is especially so in cancer, where it has been recognised since 2005 in government policy in the UK.^2^ The *Improving Outcome Guidance* issued by the National Institute for Health and Care Excellence (NICE) determined that young people aged 15–18 years receive care in a principal treatment centre (PTC) and those aged 19–24 years should have unhindered access to ‘age-appropriate’ care.^2^ The implementation of this guidance continues, accompanied by a national evaluation of TYA cancer services (BRIGHTLIGHT, NIHR PGfAR RP-PG-1209-10013). The *Improving Outcomes Guidance* also suggested an appropriate composition of the multidisciplinary team (MDT) for TYA, mirroring those proposed by the Adolescent and Young Adult Oncology Program Review Group in the USA.^3^

While suggestions have been made as to the core membership of the MDT, nothing has been proposed as to members' level of competence or expertise. An exploration among a group of experts in the USA provided a template for TYA education, mainly focusing on medical knowledge and care delivery;^4^ however, this process involved only ‘experts’ from a limited representation of the MDT and did not specify competencies that reflected the roles/tasks, and the attributes of a competent professional which may be required to care for young people. Similarly, recent guidance on the delivery of TYA cancer care in the UK has noted: “The best standard of care for teenage and young adult patients is undoubtedly provided by clinicians who have been specifically trained to care for them”.^5^ The recommendation was for professionals to participate in TYA-specific education, but no guidance was provided as to what staff type/professional roles this should involve or what level of competence professionals should aspire to.

Competence can be defined as having knowledge, skill and experience to be able to fulfil the requirements of one's professional role.^6^ Competence is often conceived as complex structuring of attributes needed for intelligent performance in specific situations and incorporates the idea of professional judgement.^7^ Within the context of realistic professional competence, it specifically brings together attributes of knowledge, attitudes, values and skills. These attributes, which jointly underlie competence, are often referred to as competencies. Competence however integrates these attributes with performance: competence is a point on the continuum of improving performance.^8^ Assessment of performance is an essential aspect of high-quality, safe and cost-effective care. Defining competencies, in terms of describing the skills which underpin job performance, is essential in education—for curriculum content development, assessment strategies and developing competency frameworks; practice development; and for management—to aid recruitment and ensure the skill mix of the workforce.^9^

While a number of competency frameworks have been developed for some professional groups in the MDT in a range of countries,^9–13^ these are either generic to the profession or specific to the care of children or adults. As it is postulated that TYA have unique needs, which requires them to have age-appropriate specialist care, there is the potential that existing competencies do not reflect the complete set of skills required to fulfil these needs. Recently, competencies for nurses caring for TYA have been published in the UK.^14^ However, to ensure uniformity in care delivery, it would be a useful exercise to develop competencies that can be applied to all professional groups represented in the MDT and also across countries to guide international professional development in teams with fledgling TYA services while still being mindful of what already exists for single professional groups.

As part of the feasibility and pilot work undertaken for BRIGHTLIGHT, a workshop (Essence of Care) was undertaken with healthcare professionals to start the process of defining the skills and attributes of health professionals working in specialist TYA cancer care. This was integrated with data collected at a Teenagers and Young Adults with Cancer (TYAC) annual meeting to provide a catalogue of key competencies. This scoping exercise provided an extensive range of competencies. The top five were identified as:

- Expertise in treating paediatric and adult cancers
- Understanding cancer
- [Delivery of] appropriate information about the disease
- Bridge between TYA need for information and parental reaction to withholding information
- Giving mutual respect^15^

## Objectives

The aim of the study was to build on this preliminary work and provide international consensus on the competencies required by healthcare professionals who provide specialist care for TYA with cancer, in order to provide the evidence to influence education, training and inform recruitment.

## Study design

**Figure 1:**
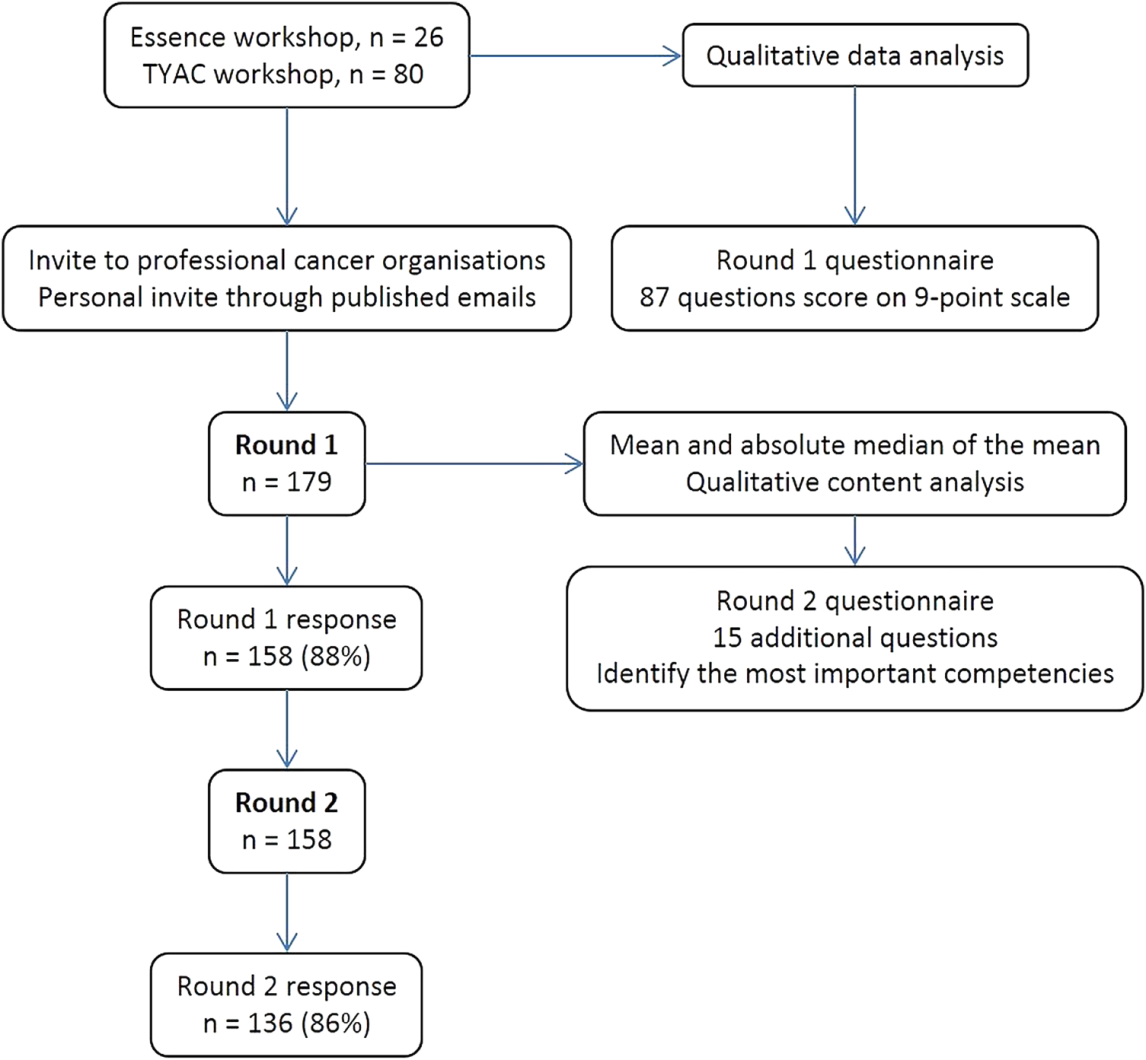
Summary of the rounds of the competency Delphi survey. Teenagers and young adults with cancer (TYAC); the professional organisation in the UK supporting members of the multidisciplinary team working with young people with cancer.

Formal or structured methods are commonly used to reach a consensus in the absence of research evidence or where there is a desire to gather opinion and initiate debate.^16^ ^17^ A commonly used formal consensus method is the Delphi technique, a method which involves two or more rounds of postal or online questionnaires. This allows involvement of large and geographically dispersed groups of participants.^18^ ^19^ A classic Delphi survey begins with an exploratory questionnaire containing mainly open-ended response questions in which to develop subsequent questionnaires.^20^ ^21^ As scoping work had previously been undertaken,^15^ the current study used a modified Delphi survey^22^ using online methods. Here, the traditional round 1 that begins with an open-ended questionnaire was replaced with a predefined list of competencies—an acceptable modification of the Delphi process reported previously.^23^ While there is no fixed number of rounds in a Delphi survey,^22^ other similar studies have suggested consensus would be reached after two rounds.^15^ ^24–27^ The study design is summarised in figure 1.

## Sample

### Definition of ‘expert’

The Delphi technique does not use a random sample representative of the target population but rather employs ‘experts’ as panel members. There is little consensus as what defines an ‘expert;’^28^ therefore, ‘expert’ for the current Delphi study was defined as any healthcare professional working in TYA cancer care for a minimum of 12 months. It was hoped that involvement of a panel of experts with a range of experiences would help to identify any country-specific competence and determine if there were any competencies that were profession specific.

### Identification of health professionals

Participants of the expert panel were recruited through purposive and snowball sampling methods to create a database reflecting the range of healthcare professionals within the MDT and also representative of the international community. In order to include as diverse a range of the membership of the MDT as possible, healthcare professionals were identified using a range of strategies:

1. Purposive
  - Requests were made to 70 international professional organisations to send an email invitation to their members.
  - A scoping exercise of literature on TYA with cancer^29^ ^30^ identified healthcare professionals working internationally in TYA cancer care. Invitations were sent to 461 email addresses extracted from publications.
  - The lead TYA clinician and senior nurse in each PTC in England were requested to send an email invitation to members of the local TYA MDT.
2. Snowball—healthcare professionals were requested to forward the invitation to all members of their TYA MDT. Specific emphasis was to include professionals other than nursing and medical staff to try and reflect the breadth of expertise and range of professionals working in the field.

### Sample size

There is no consensus as to the optimum number of participants in a Delphi survey.^22^ As a heterogeneous sample (recruiting from a number of countries, in a range of designations), it is recommended that there is a large expert panel;^31^ however, there is no consensus as to what is defined as large. The current survey therefore aimed to include all professionals who expressed an interest to participate and who fulfilled our definition of ‘expert’.

### Recruitment procedure

A strategy shown to improve recruitment and retention into a Delphi study is through personal invitation.^23^ While an open invitation was extended generally to all professionals identified using strategy 1, personal invitation was sent to those identified through authorship on publications (strategy 1B). Professionals were contacted with an introductory email and information about the survey and what participation involved. From the outset, it was made clear to professionals exactly what participation required, and over what time period, clarifying commitment if they agreed to participate. Securing that commitment to participate and ensuring professionals had a sense of ownership of the study is a further mechanism to increase response to subsequent rounds.^22^ Professionals who wanted to become members of the expert panel were asked to complete and return a registration and agreement form. The study was approved by the University Research Ethics Committee. Professionals were assured of anonymity and confidentiality, with only the research team accessing and retaining their contact details.

## Methods

### First round questionnaire

A comprehensive list of competencies was generated from a previous study,^15^ which formed the content for the first round of this Delphi survey. These were subdivided into skills (n=26; table 2), knowledge (n=18; table 3), attitudes (n=24; table 5) and communication (n=19; table 4). Communication was noted to be an integral and complex aspect of TYA cancer care^15^ and was therefore included as a separate aspect of competence. All the questions had closed-ended responses using nine-point Likert scales with the anchors: 1 = not important; 5 = of moderate importance; 9 = and extremely important. However, as the competency list was generated by healthcare professionals based in the UK, a number of open-ended questions were included to ensure the survey would accommodate the opinions of professionals in other countries. The questionnaire was administered through a web-based survey programme.

### Second round questionnaire

Only items for which there was no agreement (see analysis) were included in the round 2 questionnaire. Qualitative content analysis was used to analyse responses to open-ended questions. These were included as additional statements in the second round questionnaire, which had the same completion format as round 1. Panel members were also requested to identify five skills, areas of knowledge, communication and attitudes they considered the most important.

### Procedure

After confirming participation and on the specified date, participants were emailed an invitation to activate the round 1 questionnaire. A postal questionnaire was also available on request. In line with recommendations,^22^ ^32^ reminders were sent on a weekly basis for 3 weeks if the survey had not been returned. The survey was open for one calendar month. Only panel members who returned the round 1 questionnaire were sent the round 2 version, which was administered in the same way as round 1. It is important that as short a period as possible elapses between rounds of the survey in order to maintain interest by the panel and maximise retention,^21–23^ and therefore there was only a 2-month period between rounds.

## Data analysis

The strength/extent of agreement for each item was indicated by the median and mean absolute deviation from the median (MADM), that is, the average distance (on the nine-point Likert scale) of participants' ratings from the group's median rating.^33^ Items were ranked and reported according to the medians. Medians of 7–9 were defined as strong support, 4–6.5 as moderate and 1–3.5 as weak. MADM was calculated and the level of agreement categorised according to thirds of the MADM (low >1.41, moderate 1.08–1.41, high <1.08). These summaries were also calculated according to profession (medical doctor, nurse, other healthcare professional), and differences were determined using the χ^2^ test to compare the number of items for which there was strong agreement (consensus).

## Results

A total of 179 healthcare professionals registered to be members of the expert panel, of whom 159 (89%) returned round 1 questionnaire. Valid responses were available from 158/179 (88%) and 136/158 (86%) responded to round 2 (table 1).

**Table 1:** Characteristics of healthcare professionals who participated in rounds 1 and 2

	Round 1 n=158 n (%)	Round 2 n=136 n (%)
Professional group		
Nurse	55 (35)	50 (37)
Medical doctor	62 (39)	50 (37)
Psychology	10 (6)	10 (7)
Social worker	12 (8)	10 (7)
Allied healthcare professional	5 (3)	5 (4)
Other	11 (7)	11 (8)
Not stated	3 (2)	–
Geographical location		
Europe	85 (55)	76 (56)
Australasia	8 (5)	7 (5)
South America	2 (1)	–
North America	54 (35)	47 (35)
Asia	6 (4)	6 (4)
Unknown	3 (2)	*–*

### Round 1 survey results

There were high levels of agreement for every statement in round 1 with medians in the strong range of agreement (7–9 on the nine-point Likert scale; tables 2–5). The strength of agreement was high for most statements (MADM <1.08) but was moderate for *Cultural issues* (K1), *Environmental issues impacting young people's health* (K7), *Facilitate communication between young people* (C4), *Resolve conflicts between young people* (C5), *Resolve conflicts between young people and their families* (C7), *Act as a bridge between young people and their parents* (C10), *Facilitate care between different organisations/agencies* (C12) and *Provide career, education or training advice* (C19).

**Table 2:** The median and mean absolute deviation from the median for the skills statements in the round 1 survey (n=158)

Statement	Number (%) rating <7	Median	MADM
Being able to…			
S1. Cope emotionally	25 (16)	8	1.00
S2. Treat information sensitively	16 (11)	8	0.92
S3. Show compassion	18 (11)	8	0.95
S4. Be empathetic	6 (4)	8	0.74
S5. Be patient	16 (11)	8	0.87
S6. Balance between delivery of care and spending time with the young person	31 (20)	8	1.04
S7. Identify the impact of disease on young people's life	4 (3)	8	0.72
S8. Assess young people's social needs	15 (10)		0.84
S9. Assess young people's psychological needs	5 (3)	8	0.73
S10. Identify when care could be better delivered by other professionals or in another organisation	24 (15)	8	0.88
S11. Deliver patient-centred care	11 (7)	8	0.77
S12. Promote peer interaction	27 (17)	8	0.99
S13. Balance between patient and family-centred care	29 (18)	7	1.00
S14. Promote and enable choice	10 (6)		0.82
S15. Empower young people	7 (5)	8	0.82
S16. Provide holistic care	23 (15)	8	0.99
S17. Work in partnership with young people	12 (8)	8	0.87
S18. Be flexible in how care is delivered	13 (8)	8	0.75
S19. Provide individualised care	17 (11)	8	0.87
S20. Befriend young people but not lose professional identity	52 (33)	7	1.41
S21. Work as part of a team	12 (8)	8	0.80
S22. Provide palliative care	18 (11)	8	0.94
S23. Having tolerance	23 (15)	8	0.95
S24. Being part of a network of colleagues interested in adolescent and young adult care	24 (15)	8	0.97
S25. Be aware of professional boundaries	22 (14)	8	1.03
S26. Have excellent clinical skills	14 (9)	9	0.85

Items were rated on a nine-point Likert scale from ‘strongly agree’ to ‘strongly disagree’; median ≥7 indicated high agreement.

MADM, mean absolute deviation from the median.

**Table 3:** The median and mean absolute deviation from the median for the knowledge statements in the round 1 survey (n=158)

Statement	Number (%) rating <7	Median	MADM
Understand…			
K1. Cultural issues	38 (24)	8	1.12
K2. Issues relate to death and dying during adolescence and young adulthood	13 (8)	8	0.75
K3. Developmental issues related to emerging adulthood	13 (8)	8	0.82
K4. Family issues	18 (11)	8	0.85
K5. Issues related to risk-taking and measures to limit this	25 (16)	8	1.04
K6. Transition and how this impacts on young people at varying stages of development	26 (17)	8	1.02
K7. Environmental issues impacting young people's health	51 (32)	7	1.18
K8. The importance of peer relationships and how these may be promoted	13 (8)	8	0.78
K9. The importance of restoring normality	11 (7)	8	0.73
K10. Wider issues for young people, eg, social media	25 (16)	8	0.91
K11. Know the ethical issues related to caring for young people with cancer	16 (10)	8	0.89
K12. Have up-to-date knowledge on the policies, nationally and locally, related to caring for young people with cancer	28 (18)	8	1.01
K13. Know ways of developing coping strategies	20 (13)	8	0.89
K14. Importance to maintaining professional development	24 (15)	8	0.99
K15. Able to share knowledge	18 (11)	8	0.87
K16. Have a formal cancer-specific qualification	54 (34)	7	1.51
K17. Have a qualification specific to adolescent and young adult cancer	60 (38)	7	1.44
K18. Know how to provide age-appropriate care	13 (8)	8	0.84

Items were rated on a nine-point Likert scale from ‘strongly agree’ to ‘strongly disagree’; median ≥7 indicated high agreement.

MADM, mean absolute deviation from the median.

**Table 4:** The median and mean absolute deviation from the median for the communication statements in the round 1 survey (n=158)

Statement	Number (%) rating <7	Median	MADM
Ability to…			
C1. Act as an advocate for young people	33 (21)	8	1.05
C2. Tell young people about all aspects of their disease	27 (17)	8	1.03
C3. Liaise with other professionals on young people's behalf	15 (10)	8	0.82
C4. Facilitate communication between young people	39 (25)	8	1.17
C5. Resolve conflicts between young people	67 (42)	7	1.27
C6. Resolve conflicts between young people and health professionals	32 (20)	8	1.01
C7. Resolve conflicts between young people and their families	35 (22)	8	1.12
C8. Listen to young people's concerns	2 (1)	9	0.41
C9. Talk about difficult issues	1 (1)	9	0.49
C10. Act as a bridge between young people and their parents	41 (26)	8	1.11
C11. Allow young people time to come to their own solutions	22 (14)	8	0.85
C12. Facilitate care between different organisations/agencies	34 (22)	8	1.11
C13. Provide emotional support to young people	13 (8)	8	0.82
C14. Provide bereavement support when peers pass away	24 (15)	8	0.97
C15. Speak to young people in terms that are familiar to them while retaining a professional boundary	19 (12)	8	0.91
C16. Talk to young people about sexual issues	15 (10)	8	0.87
C17. Provide life skills support	34 (22)	8	1.05
C18. Discuss the impact of disease on aspirations	17 (11)	8	0.82
C19. Provide career, education or training advice	54 (34)	7	1.34

Items were rated on a nine-point Likert scale from ‘strongly agree’ to ‘strongly disagree’; median ≥7 indicated high agreement.

MADM, mean absolute deviation from the median.

**Table 5:** The median and mean absolute deviation from the median for the attitude statements in the round 1 survey (n=158)

Statement	Number (%) rating <7	Median	MADM
A1. Friendly and approachable	8 (5)	8	0.72
A2. Resilience	32 (21)	8	1.02
A3. Self-awareness	23 (15)	8	0.89
A4. Caring	7 (5)	8	0.73
A5. Sense of humour	19 (12)	8	0.89
A6. Be able to laugh at yourself	20 (13)	8	1.00
A7. Honesty	3 (2)	9	0.41
A8. Be positive	25 (16)	8	0.93
A9. Be relaxed	28 (18)	8	1.01
A10. Be calm	18 (11)	8	0.91
A11. Be respectful	7 (4)	9	0.59
A12. Be consistent	9 (6)	9	0.74
A13. Have energy	30 (19)	8	1.03
A14. Be motivated	16 (10)	8	0.83
A15. Ready for a challenge	23 (15)	8	0.94
A16. Open to new ideas	10 (6)	8	0.75
A17. Be creative	22 (14)	8	1.01
A18. Willing to learn	13 (8)	9	0.82
A19. Ability to learn from others	12 (8)	8	0.78
A20. Be committed to caring for young people with cancer	7 (5)	9	0.54
A21. Be passionate for working with young people	16 (10)	9	0.83
A22. Be a member of an adolescent and young adult with cancer professional body	50 (32)	7	1.41
A23. Have attention to detail	32 (21)	8	1.15
A24. Able to have a work–life balance	34 (22)	8	1.27

Items were rated on a nine-point Likert scale from ‘strongly agree’ to ‘strongly disagree’; median ≥7 indicated high agreement.

MADM, mean absolute deviation from the median.

**Figure 2:**
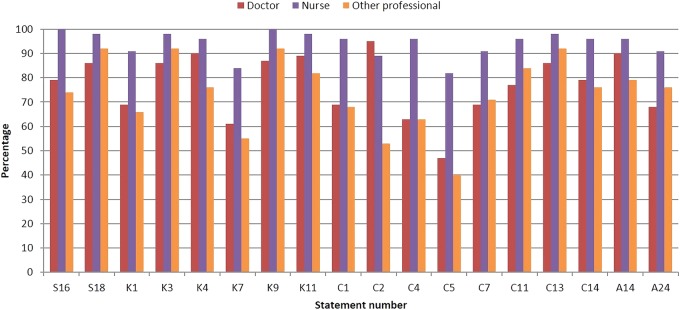
Areas of competence where there were significant differences in agreement according to professional designation (percentage of respondents who strongly agreed (scores ≥7) to statements in round 1). S16. Provide holistic care; S18. Be flexible in how care is delivered. K1. Cultural issues; K3. Developmental issues related to emerging adulthood; K4. Family issues; K7. Environmental issues impacting young people's health; K9. The importance of restoring normality; K11. Know the ethical issues related to caring for young people with cancer. C1. Act as an advocate for young people; C2. Tell young people about all aspects of their disease; C4. Facilitate communication between young people; C5. Resolve conflicts between young people; C7. Resolve conflicts between young people and their families; C11. Allow young people time to come to their own solutions; C13. Provide emotional support to young people; C14. Provide bereavement support when peers pass away. A14. Be motivated; A24. Able to have a work–life balance.

The strength of agreement was low (>1.41) for *Befriend young people but not lose professional identity* (S20), *Have a formal cancer-specific qualification* (K16), *Have a qualification specific to adolescent and young adult cancer* (K17) and *Be a member of an adolescent and young adult with cancer professional body* (A22). Comparing across professional groups, there were 18 areas of competence where there were differences in levels of agreement (figure 2).

Nurses reported more strongly that it was important to provide holistic care (χ^2^=16.69, p=0.002), have knowledge of cultural (χ^2^=11.59, p=0.02) and environmental issues impacting on health (χ^2^=11.14, p=0.03) and restoring normality (χ^2^=11.24, p=0.02). There was also greater agreement that the nursing role included being an advocate for young people (χ^2^=15.87, p<0.001), facilitating communication (χ^2^=21.46, p<0.001), resolving conflicts between young people (χ^2^=26.95, p<0.001), resolving conflicts with their families (χ^2^=9.61, p=0.05), helping young people find their own solutions for problems (χ^2^=9.38, p=0.05) and providing bereavement support (χ^2^=11.72, p=0.02). Nurses had the highest agreement among the three professional groups that it was important to have a good work–life balance (χ^2^=10.79, p=0.03).

There was lower consensus among doctors for the value of being flexible in delivering care (χ^2^=6.13, p=0.05), being aware of developmental issues related to emerging adulthood (χ^2^=10.06, p=0.04) and for providing emotional support (χ^2^=6.13, p=0.05), when compared to other elements of their role. Similarly, other professionals had less agreement on being knowledgeable about family (χ^2^=9.43, p=0.009) and ethical issues (χ^2^=11.56, p=0.02), and being able to tell young people about their disease (χ^2^=35.82, p<0.001). Other professionals reported it being less important to be motivated (χ^2^=11.05, p=0.03).

Qualitative content analysis of open-ended responses identified a number of themes for which statements had not been included in round 1 (table 6). These formed the basis of the round 2 survey.

**Table 6:** Themes identified from the open-ended responses representing issues not addressed in the round 1 survey

Section	Themes
Knowledge	Fertility Communication Non-judgemental Knowledge of research Knowledge of TYA research and issues Staff skill set
Communication	Humour Professional relationship with patient Multidisciplinary team
Skills	Knowledge of TYA development and issues TYA current interests Fertility Patient-centred care Role of TYA family Staff traits Communication Disease knowledge
Attitudes	TYA development and issues

TYA, teenagers and young adults.

### Round 2 survey results

**Figure 3:**
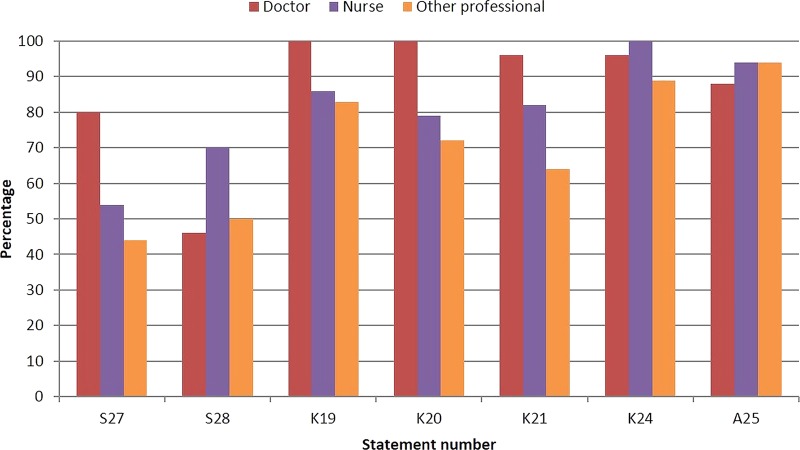
Areas of competence where there were significant differences in agreement according to professional designation (percentage of respondents who strongly agreed (scores ≥7) to statements in round 2). S27. Able to consent patients to clinical research and trials; S28. Able to address young people's concerns on spirituality appropriately. K19. Know about current therapies; K20. Know about the availability of clinical trials for this age group; K21. Know about new drugs; K24. Know about side effects of treatment and how this might be different to those experienced by children or older adults. A25. Ability to use humour appropriately when interacting with young people.

Consistent with round 1, there was strong agreement for every statement (table 7). Strength of agreement was moderate for *Able to address young*'*people's concerns on spirituality appropriately* (S28) and *Know about paediatric oncology* (K25), and low for *Able to consent patients to clinical research and trials* (S27). Again, there were differences between professional groups (figure 3). Doctors had greater agreement in the importance of being competent to consent to clinical trials (χ^2^=19.28, p=0.001), having knowledge of current therapies (χ^2^=10.57, p=0.03), available trials (χ^2^=15.48, p<0.001) and new drugs (χ^2^=16.10, p=0.003) but reported it being less important being able to use humour appropriately (χ^2^=11.65, p=0.02). Being able to address young people's spiritual needs was an aspect of competence nurses had more agreement with (χ^2^=11.00, p=0.03), while other professionals reported it being less important to know the side effects of treatment and how these differed compared with children and older adults (χ^2^=6.16, p=0.05).

**Table 7:** The median and mean absolute deviation from the median for the 15 statements in round 2 survey (n=136)

Statement	Number (%) rating <7	Median	MADM
*Skills*			
S27. Able to consent patients to clinical research and trials	53 (39)	7.00	1.54
S28. Able to address young people's concerns on spirituality appropriately	60 (44)	7.00	1.40
S29. Able to discuss sensitive subjects, eg, sexual issues, fertility	3 (2)	9.00	0.63
*Knowledge*			
K19. Know about current therapies	13 (10)	9.00	0.93
K20. Know about the availability of clinical trials for this age group	22 (16)	8.00	1.05
K21. Know about new drugs	24 (18)	8.00	0.99
K22. Know about normal physical and psychological development	9 (7)	9.00	0.75
K23. Know about impact of cancer on psychological development	7 (5)	9.00	0.75
K24. Know about side effects of treatment and how this might be different to those experienced by children or older adults	6 (4)	9.00	0.64
K25. Know about paediatric oncology	41 (30)	8.00	1.36
K26. Know about adult oncology	37 (27)	7.00	1.06
K27. Know about fertility preservation	9 (7)	9.00	0.81
K28. Know about normal adolescent physiology	8 (6)	8.00	0.82
K29. Know about the availability of psychosocial research for this age group	29 (21)	7.00	1.03
*Attitudes*			
A25. Ability to use humour appropriately when interacting with young people	20 (15)	8.00	0.90

Items were rated on a nine-point Likert scale from ‘strongly agree’ to ‘strongly disagree’; median ≥7 indicated high agreement.

MADM, mean absolute deviation from the median.

**Figure 4:**
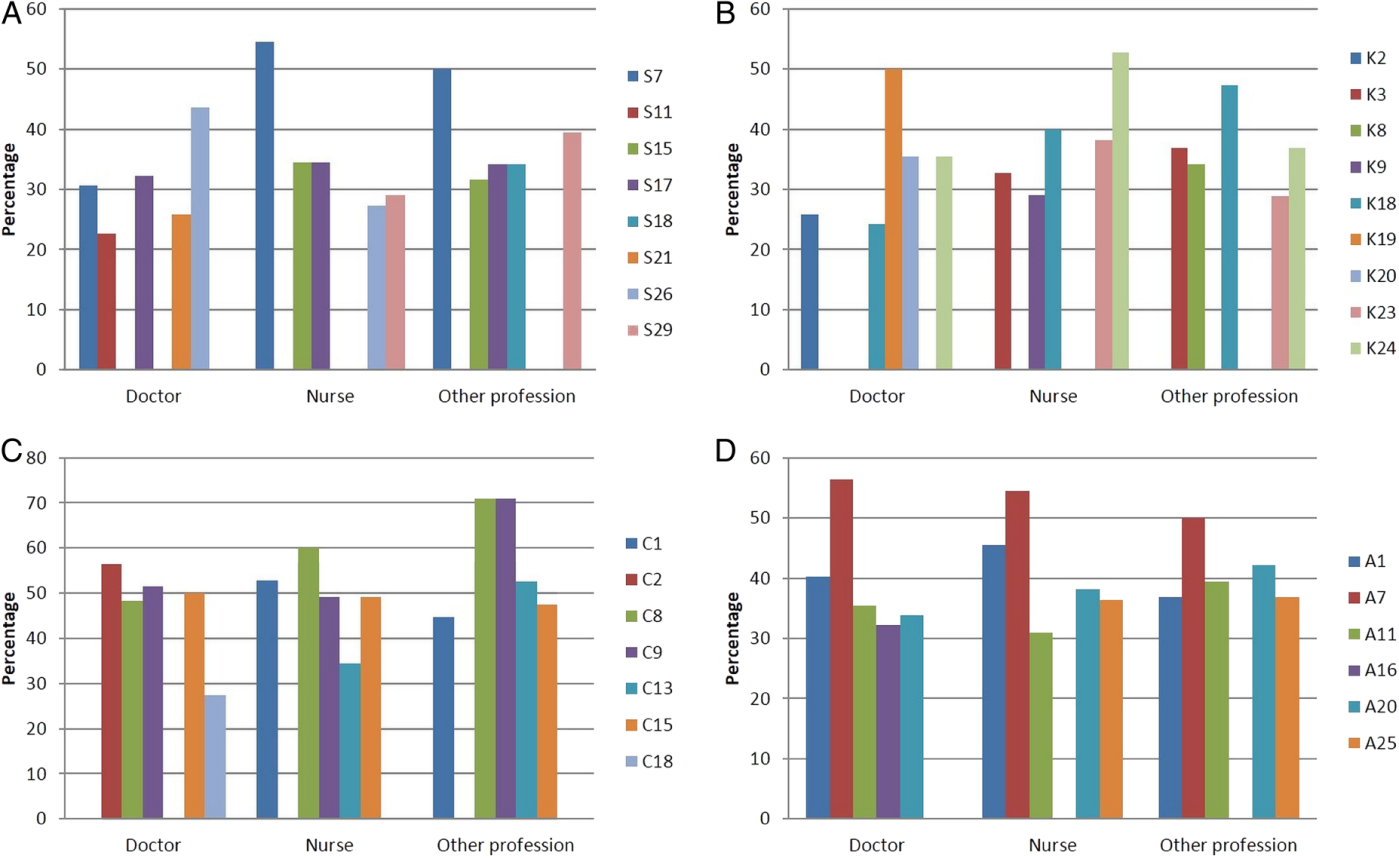
(A–D) Top five aspects of competence according to professional group. (A) Skills—S7: Identify the impact of disease on young people's life; S11: Deliver patient-centred care; S15: Empower young people; S17: Work in partnership with young people; S18: Be flexible in how care is delivered; S21: Work as part of a team; S26: Have excellent clinical skills; S29: Able to discuss sensitive subjects—for example, sexual issues, fertility. (B) Knowledge—K2: Issues relate to death and dying during adolescence and young adulthood; K3: Developmental issues related to emerging adulthood; K8: The importance of peer relationships and how these may be promoted; K9: The importance of restoring normality; K18: Know how to provide age-appropriate care; K19: Know about current therapies; K20: Know about the availability of clinical trials for this age group; K24: Know about side effects of treatment and how this might be different to those experienced by children or older adults. (C) Communication—C1: Act as an advocate for young people; C2: Tell young people about all aspects of their disease; C8: Listen to young people's concerns; C9: Talk about difficult issues; C13: Provide emotional support to young people; C15: Speak to young people in terms that are familiar to them while retaining a professional boundary; C18: Discuss the impact of disease on aspirations. (D) Attitude—A1: Friendly and approachable; A7: Honesty; A11: Be respectful; A16: Open to new ideas; A20: Be committed to caring for young people with cancer; A25: Ability to use humour appropriately when interacting with young people.

Finally, participants were asked to rate the five skills, areas of knowledge, communication and attitudes they felt to be most important. The top five areas of competence for each aspect of consensus are shown in table 8. As shown in figure 4A–D, there were aspects of competence all the professional groups agreed were important. There was most agreement regarding attitudes required for caring for young people with cancer: being friendly and approachable; being honest; being respectful; and being committed to caring for young people with cancer. Other areas of competence where there was agreement included being able to identify the impact of disease on young people's lives and working in partnership with young people; knowing how to provide age-appropriate care and the side effects of treatment and how this might be different to those experienced by children or older adults. There was agreement that key aspects of communication were being able to listen to young people's concerns, talking about difficult issues and being able to speak to young people using terms that were familiar to them while retaining a professional boundary.

**Table 8:** Top five areas of competence

Top five	Skill (n)	Knowledge (n)	Attitude (n)	Communication (n)
1	S7: Identify the impact of disease on young people's life (68; 50%)	K24: Know about side effects of treatment and how this might be different to those experienced by children or older adults (65; 48%)	A7: Honesty (84; 62%)	C8: Listen to young people's concerns (90; 66%)
2	S26: Have excellent clinical skills (53; 39%)	K18: Know how to provide age-appropriate care (55; 40%)	A1: Friendly and approachable (64; 47%)	C9: Talk about difficult issues (86; 63%)
3	S17: Work in partnership with young people (52; 38%)	K19: Know about current therapies (50; 37%)	A20: Be committed to caring for young people with cancer (58; 43%)	C15: Speak to young people in terms that are familiar to them while retaining a professional boundary (76; 56%)
4	S29: Able to discuss sensitive subjects, eg, sexual issues, fertility (43; 32%)	K23: Know about impact of cancer on psychological development (42; 31%)	A11: Be respectful (54; 40%)	C2: Tell young people about all aspects of their disease (63; 46%)
5	S11: Deliver patient-centred care (39; 29%)	K3: Developmental issues related to emerging adulthood (41; 30%)	A25: Ability to use humour appropriately when interacting with young people (52; 38%)	C1: Act as an advocate for young people (59; 43%)

## Discussion

The primary aim of this study was to reach international consensus on the competencies required by healthcare professionals who provide specialist care for TYA with cancer, in order to provide the evidence to influence education and training. By starting with a predetermined list of competencies originating from healthcare professionals in an earlier study,^15^ the traditional round 1 of a Delphi survey, referred to as item generation, was superfluous. Overall, this method enabled panellists to reach a consensus, with consistent high levels of agreement reached on all statements related to skills, knowledge, attitudes and communication. Additional statements contributing to round 2 also achieved high levels of agreement. The strength of agreement was mostly high, but where this was moderate or low, the subanalysis according to profession highlighted these as aspects that had greater agreement according to specific professional groups. Confidence in reaching consensus means we now have a comprehensive framework of competency statements that describe what healthcare professionals working with young people with cancer are required to do.

Practice guidelines described recently stated that TYA centres will need a specialist team dedicated to providing age-appropriate TYA care.^34^ This listed professional role titles, which is a helpful starting point but may not be enough if we are to deliver person-centred care to this population. In order to provide person-centred care, it is necessary to focus on the elements of care, support and treatment that matter most to patients, their families and carers.^35^ Professional boundaries may need to be challenged to have a more collaborative professional team that focuses on ‘who has the appropriate skill set for the task’ and ‘what skills are shared across professional groups’ rather than ‘which professional role is expected to do that particular task’—of most importance where there is a sharing of roles and expertise as there is in cancer care. Competency statements agreed here can be used to realise skill sets for each professional role. In practice, this will aid recruitment of professionals with the right skill set to dedicated TYA teams and used thereafter in individual performance reviews to assess ongoing competence and direct local training and educational needs. Training for care providers of this population continues to be a priority.^36^ The statements that have been developed will guide educational programmes, ensure a standard of practice, be useful in benchmarking exercises and other forms of peer review and helpful for assessment of individual and team performance.

In round 2, we asked the expert panel to identify their overall top five aspects of competence (table 8). We might consider these as essential role descriptors. Among these, many relate to the delivery of person-centred care, such as ‘*working in partnership with young people*’, ‘*listening to the concerns of young people*’, ‘*identify the impact of disease on a young person's life*’ and ‘*know how to provide age-appropriate care*’. We present them first as a composite list that combines data from the professional groups in our expert panel. Thus, we are suggesting that all professional groups would consider these competencies essential to their role. Essential might well be translated into core, that is, what is needed to practise competently in TYA cancer care. While we have described and achieved consensus on the skills, knowledge, attitudes and communication skills required, proving these attributes are evident in the skill set of a professional is a further important element, as is ensuring these attributes are applied to individual patients in a competent manner. In practice, they may be used to account for a level of practice to all stakeholders, including service users. It will also be possible to use these core competencies in the development of job descriptions, to be used at the stage of selection and interviewing. They might also instil in all professionals the concept of ‘learning to competence’ and ‘life-long learning’ so that the addition of new attributes, and the refining of skills, knowledge and attitudes, becomes a well-established feature of personal development.^37^ Self-reflection, supervision, training and clinical governance activities can be facilitated using this core set of competencies.

To work collaboratively requires professionals to have a shared professional perspective, based on an understanding of common purpose, pooling of knowledge and expertise and the facilitation of shared decision-making.^38^ ^39^ Of interest therefore in this process was the identification of differences in levels of agreement when considered across professional groups in rounds 1 and 2 (figures 2 and 3) and in the description of rating of top competencies (figure 4A–D). Of particular interest in our endeavours to deliver person-centred care, there were some key differences noted. For example, there was more agreement with nurses than doctors to address the ‘*spiritual needs of young people*’, to consider ‘*cultural issues*’ and ‘*provide holistic care*’. Of further interest, other differences might fit within current role boundaries where there was more agreement with doctors than nurses of ‘*the need to consent patients to clinical trials*’, ‘*know about current therapies*’ and ‘*know about the availability of clinical trials*’. There were only a few examples of differences between doctors, nurses and others; most striking was the greater agreement between nurses regarding ‘*resolving conflicts between young people*’ than the other two professional groups and to a lesser extent to ‘*resolve conflicts between young people and their families*’. What each professional group sees as common and what is different are of importance when describing multiprofessional competencies as they can help clarify role expectations and develop a shared understanding of which professional group might be best placed for a particular task; role conflict and team conflict can be managed more effectively where there is clarity around roles and responsibilities.^40^ In practice, the transparency of the competency statements will ensure professional roles are better described and matched to the needs of the patient population.

The main strengths of our work are that it is based on responses from an international panel of defined experts, had a good response rate and offers a framework of competencies that describe the attributes of professionals working in the field of TYA cancer care. However, some limitations also need to be recognised. First, the composition of the expert panel, with a majority of experts working in Europe or North America, with only a single respondent representing one Asian country. Our results may therefore reflect a Western perspective not applicable everywhere. Second, ‘expert’ was defined as working with TYA (or adolescent and young adult (AYA)) for a minimum of 12 months. We did not define or specify the age of the TYA/AYA population in recognition of the global variation in the lower and upper age limits. Perceptions on the importance of areas of competence may have been influenced by this varied definition that informs service configurations—for example, expertise in looking after a predominance of the younger young adults up to 24 years may be different to those of older adults aged 40 years. Third, our expert panel was determined through our approach to sampling. Emails may not have been distributed by some of the professional groups we contacted, and other experts not publishing their work may have been missed. Our survey was presented in the English language which may have prevented some individuals we contacted from participating. The composition of the expert panel may have biased the results towards the views of professionals working in countries of the ‘Western world’, where there are already well-described differences to professionals working in other countries.^41^ ^42^ Finally, while we made comparisons between professional groups, we did not look at the difference between those who had received paediatric or adult training. This may be a factor influencing perceptions of competence. However, we did not record this information; therefore, further analysis was not possible.

Despite these limitations, at this stage, we can be confident in providing a comprehensive list of multiprofessional competency statements that can:

1. Be used when designing a new TYA service and describing the composition of a clinical team
2. Inform workforce planning for new and established clinical teams
3. Direct national and international education and training
4. Be used as a benchmark against which expectations about standards can be assessed
5. Inform competency-based assessment of performance

Through the use of a locally executed modified Delphi survey, these statements can also be refined, expanded and focused to reflect more the professional training and roles fulfilled in countries other than those represented here. For countries with fledgling TYA cancer services, these statements will provide a starting point; refinements can be achieved over time as teams become more established. For established TYA cancer services, these statements could help with benchmarking performance, facilitate progression of skills and shape lifelong learning. To be most useful, we would recommend to professional groups in our field to champion their use and share developments from using these statements to be sure that the delivery of specialism-specific education and training is consistent, ensuring young people receive an equitable service wherever they are in the world.

## References

[1] Arnett JJ Emerging adulthood: a theory of development from the late teens through the twenties. Am Psychol 2000;55:469–80. 10.1037/0003-066X.55.5.469 10842426

[2] National Institute for Health and Care Excellence. Guidance on cancer services: improving outcomes in children and young people with cancer. London: NICE, 2005.

[3] Zebrack B, Bleyer A, Albritton K Assessing the health care needs of adolescent and young adult cancer patients and survivors. Cancer 2006;107:2915–23. 10.1002/cncr.22338 17103383

[4] Hayes-Lattin BM, Mathews-Bradshaw B, Siegel S Adolescent and young adult oncology training for health professionals: a position statement. J Clin Oncol 2010;28:4858–61. 10.1200/JCO.2010.30.5508 20823410

[5] Smith S, Case L, Waterhouse K A blueprint of care for teenagers and young adults with cancer. London: Teenage Cancer Trust, 2012.

[6] Bradshaw A, Merriman C Nursing competence 10 years on: fit for practice and purpose yet? J Clin Nurs 2008;17:1263–9. 10.1111/j.1365-2702.2007.02243.x 18416778

[7] Eraut M Concepts of competence. J Interprof Care 1998;12:127–39. 10.3109/13561829809014100

[8] Khan K, Ramachandran S Conceptual framework for performance assessment: competency, competence and performance in the context of assessments in healthcare—Deciphering the terminology. Med Teach 2012;34:920–8. 10.3109/0142159X.2012.722707 23039835

[9] Gibson F, Fletcher M, Casey A Classifying general and specialist children's nursing competencies. J Adv Nurs 2003;44:591–602. 10.1046/j.0309-2402.2003.02849.x 14651682

[10] Maher J, Doyle N A competence framework for nurses. Caring for patients living with and beyond cancer. London: Macmillan Cancer Support http://www.macmillan.org.uk/documents/aboutus/health_professionals/competence-framework-for-nurses.pdf (accessed 23 Apr 2016).

[11] Oncology Nursing Society. Oncology nurse navigator core competencies. Pittsburgh, PA: ONS, 2013.

[12] Ministry of Health. National professional development framework for cancer nursing in New Zealand. Wellington: Ministry of Health, 2009.

[13] Potter R, Eriksen JG, Beavis AW Competencies in radiation oncology: a new approach for education and training of professionals for radiotherapy and oncology in Europe. Radiother Oncol 2012;103:1–4. 10.1016/j.radonc.2012.03.006 22480609

[14] Royal College of Nursing. Competencies: caring for teenagers and young adults with cancer: a competence and career framework for nursing. London: Teenage Cancer Trust, 2014.

[15] Gibson F, Fern L, Whelan J A scoping exercise of favourable characteristics of professionals working in teenage and young adult cancer care: ‘thinking outside of the box’. Eur J Cancer Care) 2012;21:330–9. 10.1111/j.1365-2354.2011.01322.x 22248098

[16] Goodman CM The Delphi technique: a critique. J Adv Nurs 1987;12:729–34. 10.1111/j.1365-2648.1987.tb01376.x 3320139

[17] Keeney S, Hasson F, McKenna H Consulting the oracle: ten lessons from using the Delphi technique in nursing research. J Adv Nurs 2006;53:205–12. 10.1111/j.1365-2648.2006.03716.x 16422719

[18] Bayley EW, Richmond T, Noroian EL A Delphi study on research priorities for trauma nursing. Am J Crit Care 1994;3:208–16. 8038850

[19] Hutchings A, Raine R, Sanderson C A comparison of formal consensus methods used for developing clinical guidelines. J Health Serv Res Policy 2006;11:218–24. 10.1258/135581906778476553 17018195

[20] Hasson F, Keeney S, McKenna H Research guidelines for the Delphi survey technique. J Adv Nurs 2000;32:1008–15. 11095242

[21] Day J, Bobeva M A generic toolkit for the successful management of Delphi studies. Electron J Bus Res Methodol 2005;3:103–16.

[22] Keeney S, Hasson F, McKenna H The Delphi technique in nursing and health research. Oxford: Wiley-Blackwell, 2011.

[23] Hsu C, Sandford BA Minimizing non-response in the Delphi process: how to respond to non-response. Pract Assess Res Eval 2007;12 http://pareonline.net/getvn.asp?v=12&n=17 URL (accessed 23 Apr 2016).

[24] Gensichen J, Vollmar HC, Sonnichsen A E-learning for education in primary care—turning the hype into reality: a Delphi survey. Eur J Gen Pract 2009;15:11–14. 10.1080/13814780902864160 19353427

[25] Fehr A, Thurmann P, Razum O Expert Delphi survey on research and development into drugs for neglected diseases. BMC Health Serv Res 2011;11:312 10.1186/1472-6963-11-312 22087801 PMC3228726

[26] Liu L, Yuan C Construction of palliative care training contents in China: a Delphi study. Cancer Nurs 2009;32:446–55. 10.1097/NCC.0b013e3181ab572e 19816164

[27] Lock LR Selecting explainable nursing core competencies: a Delphi project. Int Nurs Rev 2011;58:347–53. 10.1111/j.1466-7657.2011.00886.x 21848782

[28] Baker J, Lovell K, Harris N How expert are the experts? An exploration of the concept of ‘expert’ within Delphi panel techniques. Nurse Res 2006;14:59–70. 10.7748/nr2006.10.14.1.59.c6010 17100214

[29] Taylor RM, Fern LA, Solanki A Development and validation of the BRIGHTLIGHT Survey, a patient-reported experience measure for young people with cancer. Health Qual Life Outcomes 2015;13:107 10.1186/s12955-015-0312-7 26216214 PMC4517652

[30] Taylor RM, Pearce S, Gibson F Developing a conceptual model of teenage and young adult experiences of cancer through meta-synthesis. Int J Nurs Stud 2013;50:832–46. 10.1016/j.ijnurstu.2012.09.011 23044049

[31] Skulmoski GJ, Hartman FT, Krahn J The Delphi methods for graduate research. J Inf Technol Educ 2007;6:1–21.

[32] de Leeuw ED, Hox JJ, Dillman DA International handbook of survey methodology. New York: Psychology Press, 2008.

[33] Hutchings A, Raine R, Sanderson C An experimental study of determinants of the extent of disagreement within clinical guideline development groups. Qual Saf Health Care 2005;14:240–5. 10.1136/qshc.2004.013227 16076786 PMC1744054

[34] Stark D, Bielack S, Brugieres L Teenagers and young adults with cancer in Europe: from national programmes to a European integrated coordinated project. Eur J Cancer Care 2015. 10.1111/ecc.12365 26239724

[35] Berghout M, van Exel J, Leensvaart L Healthcare professionals’ views on patient centered care in hospitals. BMC Health Serv Res 2015;15:385 10.1186/s12913-015-1049-z 26373841 PMC4572638

[36] Nass SJ, Beaupin LK, Demark-Wahnefried W Identifying and addressing the needs of adolescents and young adults with cancer: summary of an Institute of Medicine workshop. Oncologist 2015;20:186–95. 10.1634/theoncologist.2014-0265 25568146 PMC4319626

[37] Eraut M Informal learning in the workplace. Stud Contin Educ 2004;26:247–73. 10.1080/158037042000225245

[38] Nancarrow SA, Booth A, Ariss S Ten principles of good interdisciplinary team work. Hum Resour Health 2013;11:19 10.1186/1478-4491-11-19 23663329 PMC3662612

[39] Fouche C, Kenealy T, Mace J Practitioner perspectives from seven health professional groups on core competencies in the context of chronic care. J Interprof Care 2014;28:534–40. 10.3109/13561820.2014.915514 24828623

[40] Pavlish C, Brown-Saltzman K, Jakel P The nature of ethical conflicts and the meaning of moral community in oncology practice. Oncol Nurs Forum 2014;41:130–40. 10.1188/14.ONF.130-140 24578073

[41] Calaminus G, Birch JR, Hollis R The role of SIOP as a platform for communication in the global response to childhood cancer. Pediatr Blood Cancer 2013;60:2080–6. 10.1002/pbc.24728 23940113

[42] Rodriguez-Galindo C, Friedrich P, Alcasabas P Toward the cure of all children with cancer through collaborative efforts: pediatric oncology as a global challenge. J Clin Oncol 2015;33:3065–73. 10.1200/JCO.2014.60.6376 26304881 PMC4979198

